# mRNA Vaccines: Current Applications and Future Directions

**DOI:** 10.1002/mco2.70434

**Published:** 2025-10-30

**Authors:** Jianmei Li, Yixin Liu, Jie Dai, Li Yang, Feng Xiong, Jing Xia, Jing Jin, Yangping Wu, Xingchen Peng

**Affiliations:** ^1^ Department of Biotherapy, Cancer Center West China Hospital, Sichuan University Chengdu Sichuan China; ^2^ Department of Thoracic Surgery, West China Hospital, Sichuan University Chengdu Sichuan China; ^3^ Department of High Altitude Medicine, Center for High Altitude Medicine West China Hospital, Sichuan University Chengdu Sichuan China; ^4^ Department of Medical Oncology, Cancer Center, Lung Cancer Center West China Hospital, Sichuan University Chengdu Sichuan China; ^5^ Department of Pulmonary and Critical Care Medicine West China Hospital, Sichuan University Chengdu Sichuan China

**Keywords:** immune escape, mRNA vaccines, personalized vaccines, sequence optimization, targeted mRNA vaccines, vector optimization

## Abstract

Messenger RNA (mRNA) vaccines, as a novel class of biotherapeutics, leverage mRNA technology to instruct cells to produce specific antigens, thereby inducing an immune response. In recent years, significant progress has been made in applying these vaccines to infectious disease prevention and cancer treatment. Compared with traditional vaccines, mRNA vaccines offer high programmability, as well as greatly enhanced stability and immunogenicity, achieved through nucleotide modifications and advanced delivery systems such as lipid nanoparticles. However, many challenges remain in the design and delivery of mRNA vaccines, particularly for complex conditions like cancer. This review explores the latest advances and future prospects of mRNA vaccines in both infectious disease prevention and cancer therapy. It discusses the mechanisms of tumor immune escape and examines the potential of mRNA vaccines to overcome tumor immune resistance. The review also analyzes strategies for tumor vaccine design and the development of novel delivery systems, projecting the future role of mRNA vaccines in cancer therapy. By providing theoretical guidance and technical insights, this review aims to expand the development of mRNA vaccines across broader disease areas. It offers both a theoretical framework and a practical reference for researchers focused on infectious disease control and precision cancer immunotherapy. Ultimately, these insights will help advance the clinical application of next‐generation mRNA therapeutics.

## Graphical abstract

mRNA vaccines represent an innovative platform that leverages messenger RNA technology to direct cells to produce specific antigens, thereby eliciting an immune response. mRNA vaccines offer high programmability and significantly improved stability and immunogenicity through nucleotide modifications and delivery systems such as lipid nanoparticles. During the COVID‐19 pandemic, mRNA vaccines demonstrated remarkable protective efficacy and rapid development timelines. Building on this success, the technology is now being extended into cancer treatment. However, current research remains limited regarding the in vivo biodistribution, long‐term safety, and immune response mechanisms of mRNA vaccines against complex diseases like cancer. This review explores the current applications and future prospects of mRNA vaccines, from infectious disease prevention to cancer therapy.

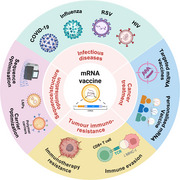

## 1 Introduction

Messenger RNA (mRNA) vaccines have evolved from a laboratory theory into a cornerstone of global health, propelled by breakthroughs in critical technological bottlenecks—particularly the optimization of mRNA modifications and delivery systems—and accelerated by the global pandemic. During the novel coronavirus (COVID‐19) pandemic, mRNA vaccines were developed with unprecedented speed and demonstrated remarkable efficacy. This not only showcased the platform's significant advantage in rapidly responding to infectious disease threats but also served as a global “proof of concept,” underscored by its exceptional effectiveness and safety profile [1]. This success has fundamentally transformed the research and development paradigm in the field of vaccines and has drawn worldwide attention to the broader applications of mRNA technology, among which cancer immunotherapy is considered one of the most promising directions.

Despite marking a milestone in infectious disease control, mRNA vaccines present a unique set of opportunities and challenges when applied to oncology. Unlike traditional vaccines that aim to prevent infectious diseases, therapeutic cancer vaccines are designed to precisely target tumor‐associated or tumor‐specific antigens (TAAs and TSAs) [2]. By selectively attacking and eliminating tumor cells expressing these antigens, such vaccines can establish long‐term efficacy through the induction of immune memory. Cancer vaccines are generally categorized into four types: tumor or immune cell vaccines, peptide vaccines, viral vector vaccines, and mRNA vaccines. Compared with other immunotherapies, cancer vaccines offer high tolerability and safety, as well as the ability to encode multiple antigens simultaneously, effectively reducing the risk of resistance during biological therapy. At present, numerous clinical trials are actively investigating the combination of personalized cancer vaccines with immune checkpoint inhibitors (ICIs) or cytokine therapies to enhance efficacy and overcome resistance to immunotherapy. These innovative combination strategies hold significant promise for the treatment of various solid tumors and metastatic cancers [3, 4, 5, 6, 7, 8].

mRNA vaccines deliver multiple TAAs or neoantigens to efficiently express target proteins in vivo, thereby activating cytotoxic T cells for precise tumor targeting [9, 10]. Their inherent advantages—including rapid development cycles, high flexibility, no risk of genomic integration, and robust activation of cellular immunity—make them an ideal platform for personalized cancer immunotherapy [11, 12, 13, 14]. Advancements in chemical modifications, codon optimization [15], and delivery systems such as lipid nanoparticles (LNPs) and polymeric carriers have significantly enhanced their translational potential [16]. During the COVID‐19 pandemic, mRNA vaccines further demonstrated their capacity for multitarget antigen expression, strong immune activation, and durable protection, accelerating their transition into cutting‐edge tumor vaccines [17, 18]. This progress holds promise for patients who have developed resistance to traditional immunotherapies.

However, seamlessly transitioning mRNA technology from infectious disease prevention to cancer treatment is not a straightforward matter of technology transfer [19, 20]. Key obstacles in current research include the immunosuppressive nature of the tumor microenvironment (TME), the identification and validation of ideal target antigens, the in vivo targeting efficiency of delivery systems, and the cost and timeliness of personalized vaccines [21, 22, 23, 24, 25, 26, 27]. Researchers worldwide are actively working to address these challenges. Early‐stage clinical trials have demonstrated encouraging antitumor activity and favorable safety profiles, but they have also revealed limitations in efficacy and highlighted substantial room for optimization [28, 29]. Therefore, it is both urgent and necessary to systematically review the latest advances in this field, clarify the core principles of key technologies, and thoroughly explore the bottlenecks and future directions of ongoing research.

This review aims to comprehensively elucidate the evolving applications of mRNA technology, ranging from infectious disease prevention to cancer therapy. We will begin by outlining the fundamental principles and advantages of mRNA vaccines, as well as their current applications in both tumor immunotherapy and infectious disease control. Next, we will examine the phenomena of immune escape and their underlying mechanisms in tumor immunotherapy, exploring strategies to overcome resistance. Finally, we will discuss future directions for mRNA cancer vaccines, including sequence design, chemical modifications, and LNP delivery systems, while emphasizing the broad application prospects of this technology in cancer therapy. This review seeks to provide researchers with an in‐depth reference on the development landscape, core challenges, and future opportunities in this rapidly advancing field.

## 2 The Basic Principles and Advantages of mRNA Vaccines

An mRNA vaccine is a synthetic immunization strategy that delivers mRNA encoding specific antigens to host cells. This approach enables host cells to express proteins from external sources, thereby eliciting targeted immune responses within the body. The mechanism not only mimics the immune response induced by natural viral infection but also offers substantial flexibility and safety. The basic principle involves the use of synthetic mRNA molecules, which typically comprise a 5′ cap structure, untranslated regions (UTRs), open reading frames (ORFs) encoding the antigen, and a poly(A) tail at the 3′ end [30, 31, 32, 33]. When mRNA is introduced into cells—usually via LNPs or other delivery systems—it is translated in the cytoplasm into the target antigen. The resulting antigen is then processed and presented through either the MHC class I or class II pathway, subsequently activating cytotoxic T lymphocytes (CTLs) (CD8⁺ T cells) and helper T lymphocytes (CD4⁺ T cells). This process ultimately generates a highly targeted and robust immune response [34, 35].

Compared with other types of vaccine platforms, mRNA vaccines offer several advantages [10, 18]. Unlike DNA vaccines or viral vector vaccines, mRNA vaccines are noninfectious and nonintegrating, thereby eliminating the potential risks of infection or insertional mutagenesis [36, 37].

In contrast to peptide vaccines, nucleic acid vaccines can encode complete tumor antigens, enabling antigen‐presenting cells (APCs) to present multiple epitopes simultaneously via both class I and class II HLA molecules. This feature allows nucleic acid vaccines to counteract tumor cell mechanisms that evade the immune system by modulating antigen expression. mRNA degradation occurs through normal cellular processes, and its half‐life in vivo can be regulated by various modifications and delivery [37, 38, 39]. mRNA vaccines are capable of delivering multiple TAAs or somatic mutations simultaneously, thereby reducing the likelihood of drug resistance during treatment [40]. The stability and translational efficiency of mRNA can be further improved through specific modifications [15]. Furthermore, the preparation cycle for mRNA vaccines is extremely [41, 42], mRNAs can be rapidly synthesized by in vitro transcription (IVT) for any target protein with a known sequence, providing valuable therapeutic windows for patients [43, 44]. While template pDNA for mRNA vaccines must be prepared via a cell culture step, this process is neither costly nor time consuming. The flexibility of mRNA vaccines is also reflected in their production process, which often allows for the rapid manufacture of variant or multivalent vaccines without significant alteration to the workflow. Additionally, mRNA vaccines possess an inherent adjuvant effect, activating immune cells to release cytokines such as tumor necrosis factor‐α and interferon‐α, thereby enhancing the immune response to antigen expression [45]. Owing to these considerable advantages, mRNAs are widely used not only in infectious disease control but also in tumor therapy, including strategies to overcome tumor immune resistance.

## 3 mRNA Vaccines are Being Used in the Field of Infectious Disease Control

In recent years, significant breakthroughs have been achieved in the development of mRNA vaccine technology for the prevention and control of infectious diseases, particularly during the COVID‐19 pandemic. This technology has demonstrated unprecedented speed and effectiveness. Compared with traditional vaccine platforms, mRNA vaccines offer several advantages, including rapid design, no requirement for cell culture, high immunogenicity, strong safety profiles, and adaptability to various pathogens. This paper reviews current research and applications of mRNA vaccines in major viral infectious diseases, including COVID‐19, influenza, respiratory syncytial virus (RSV), human immunodeficiency virus (HIV), and other pathogens.

### 3.1 COVID‐19 (SARS‐CoV‐2)

The mRNA vaccines were the first COVID‐19 vaccines to be authorized for emergency use and widely administered [46, 47]. They encode the SARS‐CoV‐2 spike protein and have demonstrated up to 95% efficacy in preventing symptomatic infection in multiple large‐scale phase III clinical trials [48]. mRNA platforms also allow for therapid updating of vaccine sequences in response to emerging viral variants. As a result, bivalent or multivalent vaccines targeting variants such as Omicron have been introduced for use as booster shots. Although waning immunity over time and reduced protection against mild infections remain challenges, mRNA COVID‐19 vaccines continue to play a central role in the global fight against outbreaks.

### 3.2 Influenza

Influenza viruses require annual updates to the influenza vaccine to match prevalent strains due to their high antigenic variability [49, 50, 51, 52, 53]. mRNA vaccine platforms are considered ideal for influenza vaccines due to their rapid production and flexibility. These vaccines work by delivering mRNAs encoding antigens, such as the influenza virus haemagglutinin (HA), prompting recipient cells to synthesize these antigens and thereby inducing an immune response [54]. Moderna's mRNA‐1010 is a tetravalent influenza mRNA vaccine covering A/H1N1, A/H3N2, and two B strains, and has entered phase II/III clinical trials [55]. Preliminary data show that mRNA‐1010 induces neutralizing antibody levels comparable to or higher than those achieved with conventional vaccines and has a favorable safety profile. Similarly, GSK's mRNA influenza vaccines have demonstrated comparable immunity against both influenza A/H1N1, A/H3N2, and two strains of influenza B in phase II trials. mRNA influenza vaccines are expected to shorten production lead times and enhance protective efficacy. The industry has shown strong enthusiasm in this area; for example, GSK recently acquired CureVac's mRNA influenza vaccine program for $430 million and is using artificial intelligence (AI) to optimize antigen design. With further improvements in antigen and delivery system optimization, mRNA influenza vaccines are anticipated to provide broader and longer‐lasting protection.

### 3.3 Respiratory Syncytial Virus

RSV is the leading cause of acute lower respiratory tract infections in infants and young children, alongside influenza viruses. It poses a significant threat to both the elderly and infants, resulting in numerous serious complications each year [56]. The development of an effective RSV vaccine has proven challenging, as previous vaccine strategies have failed due to immune response imbalances and safety concerns. However, the emergence of mRNA vaccines offers promise in overcoming the limitations of traditional vaccines, such as the risk of antibody‐dependent enhancement. Domestic company AiBio has developed a freeze‐dried RSV mRNA vaccine that utilizes proprietary base modification technology and an LNP delivery system and has already received provisional clinical licensing. This vaccine can be stored at 2–8°C for several years. In animal studies, AiBio's vaccine candidate has demonstrated significantly higher RSV‐specific IgG and neutralizing antibody levels compared with internationally marketed control vaccines, indicating strong immunogenicity. Moderna's mRNA‐1345 vaccine, which encodes the RSV fusion protein in its preF conformation, has shown 83.7% efficacy in preventing RSV‐associated lower respiratory tract disease in the elderly in a phase III clinical trial [57]. This vaccine is currently eligible for priority review under the United States Food and Drug Administration's Biologics License Application and is expected to become the first RSV vaccine based on mRNA technology [58]. The RSV vaccine market is expanding rapidly, with mRNA vaccines offering significant advantages in meeting growing demand. With enhanced delivery systems and improved stability, mRNA‐based RSV vaccines are expected to provide broader protection for infants, children, and the elderly, effectively reducing the burden of RSV infection.

### 3.4 Human Immunodeficiency Virus (HIV)

HIV vaccine development has long been hindered by high viral mutation rates, latent infection, and immune escape mechanisms [59]. Despite ongoing efforts, no effective vaccine has been approved to date. However, the success of COVID‐19 mRNA vaccines has sparked renewed interest in utilizing mRNA technology for HIV vaccine design [60, 61, 62, 63, 64]. mRNA vaccines function by delivering genetic instructions for the expression of the Env spike glycoprotein found on the surface of HIV [62], thereby eliciting both antibody and T‐cell responses against the virus. Currently, several clinical trials are underway to assess the effectiveness and safety of mRNA‐based HIV vaccines. For example, Moderna has collaborated with the NIH to develop a series of mRNA vaccines, such as mRNA‐1644, designed to induce the production of broadly neutralizing antibodies [60]. These candidates are presently being evaluated in phase I clinical trials for their immunogenicity and safety. Additionally, IAVI's G002 trial, in partnership with Moderna, is investigating a sequential mRNA immunization strategy to guide the evolution of B cells for the production of broadly neutralizing antibodies [65]. The U.S. National Institute of Allergy and Infectious Diseases has also launched the HVTN 302 trial to assess the safety and immunogenicity of three different mRNA‐based HIV vaccines [66]. These promising developments have yielded positive results in animal models and are expected to progress to more advanced clinical stages in the near future. The versatility of mRNA technology allows for the rapid testing of various antigen combinations, opening new avenues for HIV vaccine research. By combining multiple Env variants with specific modifications, researchers aim to enhance the breadth of the neutralizing antibody response against HIV [64]. With the continuous accumulation of clinical data, mRNA vaccines are anticipated to play an increasingly crucial role in HIV prevention and treatment strategies in the coming years.

### 3.5 Other Viral Diseases

In recent years, the use of mRNA vaccines to combat various viral infections has been actively explored. Cytomegalovirus (CMV), a significant cause of congenital defects, is now targeted by Moderna's mRNA‐1647 vaccine, which has entered phase III clinical trials and shows promise for preventing CMV infection [67]. mRNA vaccines for Zika virus have demonstrated the ability to induce protective immunity in animal models and have progressed to clinical trials as well. Similarly, mRNA‐based rabies vaccines are currently under clinical investigation, offering the potential for more efficient and convenient pre‐ or postexposure prophylaxis [68]. Vaccine development is also underway for herpes simplex virus using mRNA technology [69]. Additionally, mRNA vaccines against other viruses such as Ebola [70, 71] and Lassa fever [72] are in development, with researchers focusing on the genetic coding and design of key antigens. Given the lack of effective conventional vaccines for many of these viruses, mRNA technology offers the potential for rapid development of novel vaccines. mRNA platforms are highly flexible, allowing for the encoding of antigens from multiple pathogens within a single formulation, thereby enabling multivalent immunity and the creation of combination vaccines that can target multiple viruses simultaneously. The monkeypox outbreak in 2022 has further spurred research into the application of mRNA vaccines for such emerging threats [73]. With ongoing advancements in nano delivery systems and stabilizing formulation technologies—including self‐amplifying RNA and heat‐stabilized formulations—mRNA vaccines are expected to become a crucial tool in the fight against viral diseases.

## 4 mRNA Vaccines in Cancer Therapy

Among the various vaccine candidate platforms, the early application of mRNA was limited by instability, low efficiency, and excessive immunogenicity. However, the successful development of the SARS‐CoV‐2 mRNA vaccine overcame these technological bottlenecks in vaccine preparation. This breakthrough has enabled the rapid, economical production of tumor mRNA vaccines with improved stability and efficiency. In this context, we will discuss the application of mRNA vaccines in tumor immunotherapy, as well as current advances in targeted and personalized mRNA vaccines.

### 4.1 mRNA Vaccines in Tumor Immunotherapy

**FIGURE 1:**
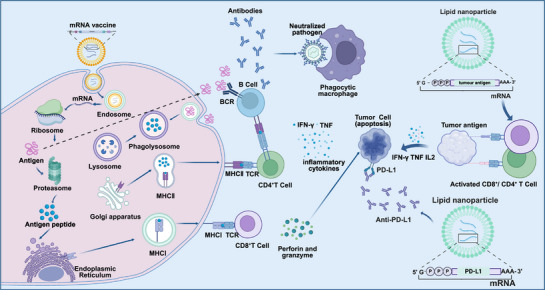
mRNA‐based vaccine mode of action. mRNA is taken up by antigen‐presenting cells and peptides are loaded on MHC class I for antigen‐specific CD8^+^ T‐cell activation. Extracellular proteins are cross‐presented on MHC class I or loaded on MHC class II for CD4^+^ T‐cell activation. CD4^+^ T cells can coactivate protein‐specific B cells, and B cells can activate CD4^+^ T cells after B‐cell receptor‐mediated antigen internalization. Created in BioRender.com.

The application of mRNA vaccines in tumor immunotherapy primarily relies on encoding TAAs or TSAs to activate the immune system for targeted attacks on tumor cells. These vaccines efficiently stimulate APCs in vivo, promoting a synergistic effect between innate and adaptive immune responses. In clinical practice, mRNA vaccines are classified as either prophylactic or therapeutic: prophylactic vaccines aim to prevent tumor development in high‐risk populations by encoding tumor antigens, while therapeutic vaccines target patients with existing tumors, eliciting immune responses to attack and eliminate cancer cells [35]. In recent years, mRNA tumor vaccines have demonstrated significant promise in clinical trials for various cancer types. Sahin et al. [74] found that mRNA vaccines induced a strong immune response in patients with melanoma, which was notably enhanced when combined with ICIs. Similarly, Yuan et al. [75] showed that personalized vaccines targeting RNA mutations triggered multspecific and therapeutic immune responses against cancer. Furthermore, a review by Miao et al. [35] confirmed the promising application of mRNA vaccines in treating a range of aggressive solid tumors, such as non‐small cell lung cancer (NSCLC), colorectal cancer, and melanoma. mRNA vaccines are efficiently delivered to target cells using LNP‐based vectors. Once inside the cell, mRNA is internalized into endosomes. Protonation of ionizable lipids in the acidic endosomal environment promotes membrane fusion, leading to the release of mRNA into the cytoplasm, where it is translated into protein. These proteins are degraded by the proteasome into antigenic peptides, which are then presented on MHC molecules to CD8⁺ T cells, thereby activating cell‐mediated immune responses [76]. Pan et al. [77] demonstrated that nanomaterial‐based delivery systems significantly enhance the immunogenicity of mRNA vaccines and exert antitumor effects via the Toll‐like receptor 4 signaling pathway. mRNA vaccines activate APCs such as dendritic cells (DCs) and macrophages, enabling them to process exogenous antigens and present them through MHC molecules. Cells display exogenous antigens to CD4⁺ T cells via MHC‐II and facilitate cross‐presentation to CD8⁺ T cells via MHC‐I. Mature DCs secrete proinflammatory and immunostimulatory cytokines—including IL‐12, IL‐23, and IL‐1β and subsequently migrate to tumor‐draining lymph nodes [78]. Zhou et al. [79] reported that incorporating a STING agonist in mRNA vaccine design can substantially increase the effectiveness of cancer immunotherapy, promoting immune cell activation and antitumor responses through intelligently designed nanovaccines. Peptides presented on DC surface MHC molecules interact with T cell receptors (TCRs), activating naïve CD8⁺ T cells and differentiating them into CTLs under the combined influence of multiple receptors and cytokines. These CTLs can infiltrate tissues and target tumor cells, exerting potent antitumor effects [80]. A study by Melamed et al. [81] showed that mRNA delivered via ionizable LNPs can be efficiently targeted to pancreatic β‐cells to induce robust CTL responses. Importantly, mRNA vaccines are also capable of inducing memory T cell formation, enabling rapid and long‐lasting protection upon re‐exposure to the same antigen. He et al. [82] highlighted the potential of mRNA cancer vaccines in inducing durable immune memory. Figure 1 illustrates the mechanisms of mRNA vaccines in tumor immunotherapy.

### 4.2 Targeted mRNA Vaccines

Tumor mRNA vaccines are highly valued for their precise target selection, as tumor antigens recognized by T lymphocytes are essential for the efficacy of cancer vaccines [83]. Ideal antigens for cancer vaccines should be highly immunogenic, expressed exclusively in cancer cells (but not in normal cells), and crucial for cancer cell survival [84]. Tumor antigens are generally categorized as TAAs and TSAs [85, 86], also known as tumor‐sharing antigens [87, 88]. Targeted mRNA vaccines offer advantages such as high specificity and low side effects, thereby minimizing damage to normal cells and reducing adverse reactions [77]. Mixtures of mRNA vaccines encoding multiple TAAs have been tested in several clinical trials for the treatment of metastatic melanoma. For example, BNT111, an mRNA vaccine encoding four TAAs (NY‐ESO‐1, MAGE‐A3, tyrosinase, and TPTE), has demonstrated efficacy as immunotherapy for patients with checkpoint inhibitor (CPI)‐refractory melanoma, highlighting the utility of nonmutated shared tumor antigens in cancer vaccination [74]. Researchers are also exploring targets that activate immune checkpoints to enhance the efficacy of mRNA vaccines. For instance, STING agonist‐enhanced mRNA vaccines improve antigen presentation and boost antitumor immune responses by activating the STING pathway in vivo [79]. Additionally, targeted mRNA vaccines can be combined with other immunotherapies, such as CPIs, to further enhance antitumor immune effects [89]. In clinical applications, targeted mRNA vaccines have shown promising efficacy across various malignancies. Although a study (NCT02410733) did not observe increased patient responses with the combination of anti‐PD‐1 therapy and the TAA vaccine FixVac compared with FixVac alone, the potential for combining mRNA vaccines with ICIs in oncology cannot be excluded [75]. In melanoma patients, direct injection of a protective mRNA induced robust T‐cell responses and significantly reduced tumor burden. In prostate cancer patients, a self‐adjuvanted mRNA vaccine effectively activated the immune system and significantly prolonged patient survival [90]. Despite the great potential of targeted mRNA vaccines in tumor therapy, their development and application face several challenges. The heterogeneity and variability of tumor antigens complicate target selection, and the efficiency and stability of mRNA vaccine delivery systems require further improvement. To address these challenges, researchers are actively developing new antigen screening methods and delivery systems to improve the efficacy and safety of targeted mRNA vaccines [75]. Table 1 provides a summary of recent clinical trials involving targeted mRNA vaccines.

**TABLE 1:** Clinical trials of targeted mRNA vaccines.

NCT number	Status	Disease	Sponsor	Phase	Start date	Last update posted
NCT05714748	Recruiting	Malignant tumors	West China Hospital	Phase 1	2022/11/18	2023/02/06
NCT05738447	Recruiting	Liver cancer hepatocellular carcinoma	West China Hospital	Phase 1	2023/02/15	2023/02/22
NCT03948763	Completed	Neoplasms carcinoma non‐small cell lung pancreatic neoplasms colorectal neoplasms	Merck Sharp & Dohme LLC	Phase 1	2019/06/26	2022/09/07
NCT03948763	Completed	Neoplasms carcinoma non‐small cell lung pancreatic neoplasms	Merck Sharp & Dohme LLC	Phase 1	2019/06/26	2022/09/07
NCT06019702	Recruiting	Digestive system neoplasms	Sir Run Run Shaw Hospital	Phase 1	2023/09/08	2023/09/11
NCT06026800	Recruiting	Digestive system neoplasms	Sir Run Run Shaw Hospital	Phase 1	2023/09/08	2023/09/11
NCT06026774	Recruiting	Digestive system neoplasms	Sir Run Run Shaw Hospital	Phase 1	2023/09/08	2023/09/11
NCT00890032	Completed	Recurrent central nervous system neoplasm	John Sampson	Phase 1	2009/09/01	2016/10/17
NCT00890032	Completed	Recurrent central nervous system neoplasm	John Sampson	Phase 1	2009/09/01	2016/10/17
NCT01278940	Completed	Malignant melanoma	Oslo University Hospital	Phase 1/2	2002/03/01	2016/08/15
NCT01278914	Completed	Prostate cancer	Oslo University Hospital	Phase 1/2	2002/02/01	2023/05/08
NCT03323398	Terminated	Relapsed/refractory solid tumor malignancies or lymphoma ovarian cancer	ModernaTX, Inc.	Phase 1/2	2017/08/15	2024/07/31
NCT03739931	Active, not recruiting	Relapsed/refractory solid tumor malignancies or lymphoma triple negative breast cancer, HNSCC, non‐Hodgkins, urothelial cancer, immune checkpoint refractory melanoma, and NSCLC lymphoma	ModernaTX, Inc.	Phase 1	2018/11/27	2024/05/17
NCT00890032	Completed	Recurrent central nervous system neoplasm	John Sampson	Phase 1	2009/09/01	2016/10/17
NCT00890032	Completed	Recurrent central nervous system neoplasm	John Sampson	Phase 1	2009/09/01	2016/10/17
NCT01278940	Completed	Malignant melanoma	Oslo University Hospital	Phase 1/2	2002/03/01	2016/08/15
NCT01278914	Completed	Prostate cancer	Oslo University Hospital	Phase 1/2	2002/02/01	2023/05/08
NCT03323398	Terminated	Relapsed/refractory solid tumor malignancies or lymphoma ovarian cancer	ModernaTX, Inc.	Phase 1/2	2017/08/15	2024/07/31
NCT03739931	Active, not recruiting	Relapsed/refractory solid tumor malignancies or lymphoma triple negative breast cancer, HNSCC, non‐Hodgkins, urothelial cancer, immune checkpoint refractory melanoma, and NSCLC lymphoma	ModernaTX, Inc.	Phase 1	2018/11/27	2024/05/17
NCT01066390	Completed	Melanoma	Bart Neyns	Phase I	2009/12/01	2014/05/06
NCT05264974	Recruiting	Melanoma	University of Florida	Phase 1	2024/11/01	2024/10/29
NCT04932863	Unknown	Neoplasms cancer, treatment‐related	Ente Ospedaliero Ospedali Galliera	Observational	2021/03/15	2021/06/21
NCT00961844	Terminated	Metastatic malignant melanoma	Steinar Aamdal	Phase 1/2	2009/08/01	2021/02/26
NCT00204516	Completed	Malignant melanoma	University Hospital Tuebingen	Phase 1/2	2007/04/01	2013/01/16
NCT00626483	Completed	Malignant neoplasms brain	Gary Archer Ph.D.	Phase 1	2007/04/24	2021/03/09
NCT05028374	Completed	Multiple myeloma AL amyloidosis chronic lymphocytic leukemia	Barbara Ann Karmanos Cancer Institute	Phase 2	2021/08/17	2023/12/20
NCT06156267	Not yet recruiting	Pancreatic cancer	Fudan University	Early‐Phase 1	2024/01/01	2023/12/05
NCT01153113	Withdrawn	Metastatic prostate cancer	University of Florida	Phase 1/2	2008/01/01	2011/12/02
NCT03164772	Completed	Metastatic non‐small cell lung cancer NSCLC	Ludwig Institute for Cancer Research	Phase 1/2	2017/12/20	2022/10/10
NCT05938387	Recruiting	Glioblastoma	CureVac	Phase 1	2023/06/01	2024/08/15
NCT04163094	Terminated	Ovarian cancer	University Medical Center Groningen	Phase 1	2019/11/25	2023/06/29
NCT04573140	Recruiting	Adult glioblastoma high grade glioma WHO grade III or IV malignant glioma	University of Florida	Phase 1/2	2021/10/26	2024/09/25
NCT04382898	Terminated	Prostate cancer	BioNTech SE	Phase 1/2	2019/12/19	2024/05/17
NCT03788083	Recruiting	Breast cancer female early‐stage breast cancer	Universitair Ziekenhuis Brussel	Phase 1	2018/11/12	2022/05/19

### 4.3 Personalized mRNA Vaccines

**FIGURE 2:**
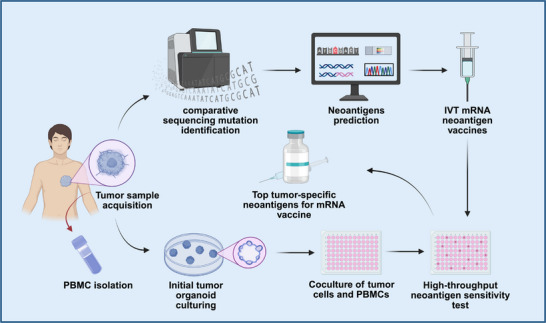
Illustration of the pipeline for synthesizing mRNA vaccines by integrating high‐throughput sequencing for patient neoantigen prediction with organoid screening. Created with BioRender.com.

Personalized mRNA cancer vaccines represent a cutting‐edge cancer therapy that works by stimulating the immune system to precisely target cancer cells using specific antigens found in a patient's tumor [91]. As cancer cells develop, they accumulate mutations that give rise to unique protein sequences—neoantigens—not present in normal cells [92]. These neoantigens are processed by the proteasome and displayed on the cell surface by MHC molecules, where they can be recognized by TCRs. Because these neoantigens are unique to each patient, they present both challenges and opportunities for personalized immunotherapy [93]. Clinical trials have demonstrated that personalized mRNA cancer vaccines are safe and well‐tolerated [94, 95]. Companies like BioNTech and Moderna have made significant progress in this field, developing personalized mRNA vaccines that have shown promising antitumor effects in clinical trials [96]. For example, Moderna's mRNA‐4157 vaccine, which can encode up to 34 neoantigens within LNPs, has demonstrated positive effects in phase I trials for solid tumors, both as a monotherapy and in combination with pembrolizumab, and is currently in phase II trials. Similarly, BioNTech's BNT122 vaccine encodes up to 20 patient‐specific neoantigens using its mRNA personalized cancer vaccine platform. This vaccine has been shown to induce neoantigen‐specific T cell responses and clinically meaningful responses in a phase 1a/1b trial and is currently being evaluated in a phase II clinical trial for colorectal cancer (NCT04486378). Sahin et al. [74] further elucidated how personalized RNA mutant vaccines can stimulate multispecific therapeutic immune responses to effectively combat cancer. These vaccines successfully activated patients’ immune systems by encoding individualized tumor neoantigens. Meanwhile, C. Pollard et al. highlighted the importance of predicting and identifying neoantigens using advanced technologies such as whole exome sequencing, RNA sequencing, and mass spectrometry. These technologies have not only streamlined vaccine production but have also improved the potential effectiveness of such vaccines [97]. One of the main challenges of personalized mRNA cancer vaccines lies in the prediction and identification of neoantigens. Currently, many neoantigen prediction methods rely on machine learning‐based algorithms [98]. mRNA vaccines are generated after candidate neoantigens are identified from tumor tissue by sequencing‐based prediction algorithms or microfluidic chip experiments (Figure 2). Personalized mRNA vaccines can also be combined with other treatments, such as chemotherapy and radiotherapy, to achieve synergistically enhanced therapeutic effects [99, 100]. In clinical practice, personalized mRNA vaccines have shown encouraging results in the treatment of various malignancies. For instance, in a clinical trial involving patients with gastrointestinal cancers, personalized mRNA vaccines successfully induced specific T cell responses and significantly reduced tumor burden [94]. In studies on melanoma patients, these vaccines effectively activated the immune system and significantly prolonged patient survival [74]. Although personalized mRNA vaccines show great potential in oncology, there are still several challenges in their development and application. The process of preparing personalized vaccines is complex and costly. In addition, the delivery efficiency and stability of mRNA vaccines need to be further optimized. To overcome these obstacles, researchers are actively exploring new antigen screening methods and novel delivery systems, with the aim of improving the efficacy and safety of personalized mRNA vaccines [101, 102, 103]. Table 2 summarizes the clinical trials of personalized mRNA vaccines in recent years.

**TABLE 2:** Clinical trials of personalized mRNA vaccines.

NCT number	Status	Disease	Sponsor	Phase	Start date	Last update posted
NCT06496373	Recruiting	Pancreatic cancer recurrent	Ruijin Hospital	Phase 1	2024/04/01	2024/07/11
NCT05916248	Recruiting	Advanced solid tumor	Ruijin Hospital	Phase 1	2023/05/18	2023/10/10
NCT05916261	Recruiting	Advanced pancreatic cancer	Ruijin Hospital	Early phase 1	2023/04/26	2023/10/26
NCT02709616	Completed	Glioblastoma	Guangdong 999 Brain Hospital	Phase 1	2016/03/01	2022/05/27
NCT02808416	Completed	Brain cancer neoplasm metastases	Guangdong 999 Brain Hospital	Phase 1	2016/03/01	2022/05/27
NCT02808364	Completed	Glioblastoma	Guangdong 999 Brain Hospital	Phase 1	2016/03/01	2022/05/27
NCT05761717	Not yet recruiting	Postoperative hepatocellular carcinoma	Shanghai Zhong shan Hospital	Not applicable	2023/04/20	2023/03/09
NCT03480152	Terminated	Melanoma colon cancer gastrointestinal cancer genitourinary cancer hepatocellular cancer	National Cancer Institute (NCI)	Phase 1/2	2018/05/18	2020/06/02
NCT06141369	Recruiting	Adrenal cortical carcinoma medullary thyroid cancer thymic neuroendocrine carcinoma pancreatic neuroendocrine tumor	Shanghai Jiao Tong University School of Medicine	Not applicable	2024/01/13	2024/01/26
NCT05359354	Recruiting	Solid tumor	YueJuan Cheng	Not applicable	2022/09/01	2023/05/16
NCT02035956	Completed	Melanoma	BioNTech RNA Pharmaceuticals GmbH	Phase 1	2013/12/01	2020/01/18
NCT03897881	Recruiting	Melanoma	ModernaTX, Inc.	Phase 2	2019/07/18	2024/06/03
NCT03380871	Completed	Carcinoma, non‐small cell lung cancer, non‐squamous non‐small cell neoplasm of lung	BioNTech US Inc.	Phase 1	2018/05/04	2021/02/26
NCT06026774	Recruiting	Digestive system neoplasms	Sir Run Run Shaw Hospital	Phase 1	2023/09/08	2023/09/11
NCT06026800	Recruiting	Digestive system neoplasms	Sir Run Run Shaw Hospital	Phase 1	2023/09/08	2023/09/11
NCT06497010	Recruiting	Advanced solid tumor	The Affiliated Hospital of Guizhou Medical University	Early phase 1	2024/08/01	2024/08/01
NCT05227378	Not yet recruiting	Gastric cancer	Shen Lin	Not applicable	2022/03/01	2022/02/07
NCT05981066	Recruiting	Advanced hepatocellular carcinoma	Peking Union Medical College Hospital	Not applicable	2023/07/10	2023/08/08
NCT03164772	Completed	Metastatic non‐small cell lung cancer NSCLC	Ludwig Institute for Cancer Research	Phase 1/2	2017/12/20	2022/10/10
NCT06326736	Recruiting	Pancreatic cancer	Jinling Hospital, China	Early phase 1	2024/04/01	2024/03/22
NCT03908671	Recruiting	Esophageal cancer non‐small cell lung cancer	Stemirna Therapeutics	Not applicable	2019/10/18	2023/04/13
NCT05949775	Not yet recruiting	Advanced malignant solid tumors	Stemirna Therapeutics	Not applicable	2023/07/20	2023/07/18
NCT05198752	Unknown	Solid tumor	Stemirna Therapeutics	Phase 1	2022/03/18	2022/10/17
NCT03468244	Recruiting	Advanced esophageal squamous carcinoma gastric adenocarcinoma pancreatic adenocarcinoma colorectal adenocarcinoma	Changhai Hospital	Not applicable	2018/05/01	2019/02/26
NCT06195384	Recruiting	Solid tumor	Second Affiliated Hospital of Guangzhou Medical University	Phase 1	2024/05/08	2024/06/26
NCT05192460	Recruiting	Gastric cancer esophageal cancer liver cancer	Jianming Xu	Not applicable	2022/03/28	2023/03/07
NCT06295809	Active, not recruiting	Carcinoma, squamous cell skin neoplasms	Merck Sharp & Dohme LLC	Phase 2	2024/04/18	2026/03/05
NCT06307431	Active, not recruiting	Renal cell carcinoma	Merck Sharp & Dohme LLC	Phase 3	2024/04/10	2032/06/08
NCT05933577	Active, not recruiting	Melanoma	Merck Sharp & Dohme LLC	Phase 2	2023/07/19	2030/09/26
NCT06961006	Not yet recruiting	Malignant melanoma	Merck Sharp & Dohme LLC	Phase 3	2025/06/10	2031/09/05
NCT06077760	Recruiting	Non‐small cell lung cancer	Merck Sharp & Dohme LLC	Phase 2	2023/12/06	2035/12/21

## 5 The Potential for mRNA Vaccines to Overcome Tumor Immune Resistance

The issue of drug resistance in tumor cells has long posed a significant challenge in cancer treatment. Drug resistance not only diminishes the effectiveness of existing therapies but also increases the complexity and difficulty of disease management. In recent years, mRNA vaccines have shown promising advancements in tumor therapy, particularly in addressing immune escape and drug resistance. These vaccines work by delivering mRNAs encoding tumor antigens into cells, thereby activating APCs and stimulating specific T and B cell responses. This technology can be tailored to target tumor neoantigens and overcome drug resistance by modulating the immune microenvironment. In this discussion, we will explore the current challenges of immunotherapeutic resistance, the mechanisms underlying immune escape in tumor immunotherapy, and the potential of mRNA vaccines to overcome these barriers.

### 5.1 The Challenge of Immunotherapy Resistance

The phenomenon of drug resistance in tumor immunotherapy can be classified into three types: primary, adaptive, and acquired resistance [104, 105]. Primary resistance occurs when the initial treatment is ineffective. Acquired resistance develops when tumor cells regain a survival advantage after treatment, often through genetic mutations [4]. Adaptive resistance involves tumor cells evading immune attack, with one well‐known mechanism being the expression of B7‐H1 in response to IFN‐γ released by T cells within the TME [106]. Recent studies have reported various immune resistance phenomena, such as an initial favorable response to ICI therapy that becomes difficult to sustain, resulting in reduced therapeutic effectiveness over time [107]. For example, research by He et al. [27] demonstrated that, in hepatocellular carcinoma, serum amyloid A promotes glycolysis in neutrophils, rendering them resistant to anti‐PD‐1 blockade therapy. This finding underscores the critical role of specific factors within the TME in the mechanisms underlying ICI resistance, highlighting the need to focus on modulating the TME when developing strategies to overcome drug resistance. In addition to the TME, intrinsic factors within tumor cells themselves also play a significant role in resistance [108]. Furthermore, drug resistance to CAR‐T cell therapy is closely associated with immunosuppression in the TME. Tumors can evade immune attack by modulating cellular and signaling components in their environment, such as downregulating MHC molecule expression to escape CAR‐T cell recognition, ultimately leading to treatment failure [109]. Tumor heterogeneity is strongly linked to acquired drug resistance, affecting the recognition and cytotoxic ability of CAR‐T cells [6]. In contrast, resistance to NK cell therapy is mainly attributed to immunosuppression within the TME and adaptive alterations in tumor cells. Weber et al. [110] discovered that myeloid‐derived suppressor cells (MDSCs) inhibit NK cell activity in the TME and promote the generation of regulatory T cells (Tregs), resulting in resistance to ICIs. Additionally, genetic mutations and upregulation of metabolic pathways in tumor cells—such as the activation of oxidative phosphorylation and DNA repair pathways as investigated by Memon et al. [6] allow these cells to survive and proliferate under immune stress. Sharma et al. [4] further noted that tumor cells can evade NK cell recognition by modulating their surface antigen expression, for instance, by downregulating activating receptor ligands or upregulating inhibitory receptor ligands. Finally, DCs play a key role in tumor immunotherapy by activating T cell responses. However, drug resistance may reduce the effectiveness of DC‐based therapies. Tumor cells can escape immune surveillance by decreasing antigen presentation, altering tumor antigens, or modulating the expression of immune checkpoint molecules, thereby influencing the efficacy of DC therapy. To counter drug resistance, researchers have explored combination strategies, such as pairing DC therapy with anti‐PD‐1 or anti‐CTLA‐4 antibodies, to enhance antitumor immune responses and reduce resistance [111].

### 5.2 Mechanisms Associated with Immune Evasion in Tumor Immunotherapy

Tumor cells can evade immune system recognition by decreasing the expression of tumor antigens. These antigens, which include TSAs and TAAs, play a crucial role in enabling the immune system to detect and attack tumor cells. Tumor cells employ various strategies to downregulate the expression of these antigens, thereby reducing their chances of being identified and targeted by T cells. One such strategy involves acquiring genetic mutations that inactivate or alter the genes encoding tumor antigens. These mutations can result in the complete loss or structural modification of tumor antigens, making them less recognizable to TCRs. Additionally, mutations in the coding regions of tumor antigen genes may cause changes or truncations in protein sequences, further hindering recognition by T cells. Such mechanisms contribute to immune evasion and present significant challenges to the effectiveness of immunotherapy [112]. Understanding these processes offers valuable insight into the development of resistance to immune‐based treatments.

#### 5.2.1 Altering MHC I Molecular Expression

MHC class I molecules play a crucial role in the tumor immune response by displaying intracellular antigenic peptides on the surface of tumor cells, thereby providing targets for T cells to recognize and bind to, which stimulates an immune reaction. Unfortunately, tumor cells often adopt the strategy of downregulating MHC class I molecule expression to evade immune surveillance; this has become a common method by which tumors escape immune attack. Kalbasi and Ribas [108] revealed a mechanism whereby tumor cells resist cancer immunotherapy by reducing the expression of HLA molecules. In addition, mutations affecting MHC class I molecules represent another mechanism for tumors to evade immune recognition. Using advanced computational tools, McGranahan et al. [113] identified loss of heterozygosity in HLA genes among patients with NSCLC and found that approximately 40% of patients exhibited this phenomenon, resulting in diminished neoantigen presentation and further facilitating immune escape. Mutations in the β2‐microglobulin (β2m) gene constitute another key immune evasion mechanism. In studies of lung cancer patients resistant to PD‐1 therapy, Gettinger et al. [114] found that certain tumors exhibited loss of heterozygosity for β2m, resulting in a lack of HLA class I molecule expression on the cell surface, while others showed downregulation of β2m expression. These mutations not only render MHC‐I molecules unstable, but also prevent efficient presentation of neoantigens to tumor‐infiltrating lymphocytes, thereby allowing tumor cells to evade immune surveillance. Furthermore, activation of the β‐catenin signaling pathway in tumor cells can also alter MHC molecule expression and suppress the antitumor immune response [115, 116]. The activation of this signaling pathway not only inhibits the function of effector T cells but also promotes the accumulation of immunosuppressive cells, thereby enhancing the tumor's ability to evade the immune system. D'Urso et al. [117] have further indicated that certain melanoma cells are deficient in the expression of the β2m gene, resulting in the loss of HLA class I antigens and allowing these cells to escape immune recognition.

#### 5.2.2 Reduced Immunogenicity

Tumor cells can reduce their immunogenicity by secreting immunosuppressive factors such as TGF‐β and IL‐10. These factors inhibit T cell activity and promote the proliferation of Tregs, thereby suppressing the immune response. Additionally, cells within the TME—such as tumor‐associated macrophages (TAMs) and MDSCs—further facilitate immune evasion by secreting these same immunosuppressive factors. The resulting immunosuppressive microenvironment restricts antitumor immunity through the combined action of factors secreted by tumor cells, immune cells, and stromal cells. For example, elevated levels of IL‐8 may reflect a particularly unfavorable TME that impedes antitumor immunity in preclinical models as well as certain human cancers [118]. According to Vesely and colleagues [3], tumor cells inhibit the function of effector T cells and promote the accumulation of immunosuppressive cells by secreting factors such as TGF‐β and IL‐10, a strategy that enables tumors to grow and metastasize while escaping immune surveillance. Furthermore, B cell‐derived IL‐35 mediates the exclusion of CD8+ T cells in pancreatic cancer, promoting immune escape through a STAT3‐dependent mechanism [119]. Cytokine secretion not only impairs effector T cell function but also encourages the accumulation of other immunosuppressive cells, thereby intensifying tumor immune escape. Tregs [120], MDSCs [121], and TAMs [122], all suppress antitumor immune responses in preclinical cancer models. Notably, infiltration of Tregs into the TME has been observed in various cancer types [123, 124, 125, 126]. It has been suggested that the modulation of the immune response by anti‐CTLA‐4 therapies may be attributed to their targeting of Tregs [127, 128, 129, 130, 131], Sharma et al. [4] have demonstrated that Tregs directly inhibit effector T cell activity by secreting inhibitory cytokines such as IL‐10 and IL‐35, thereby perpetuating tumor immune escape. High levels of Treg infiltration in many tumors are closely associated with immune evasion [4]. MDSCs are heterogeneous myeloid cells recruited to the TME that possess immunosuppressive functions [121]. Weber et al. [110] suggested that MDSCs accumulate in the TME and suppress the activity of effector T cells through the secretion of immunosuppressive factors such as IL‐10 and TGF‐β. Additionally, MDSCs can further impair the immune response by inducing T cell apoptosis and inhibiting T cell proliferation [110]. TAMs are considered predictors of patient prognosis, and targeting these cells remains a prominent area of clinical research [122]. Leal and John [8] have suggested that TAMs promote tumor growth and immune escape by secreting cytokines and chemokines such as IL‐10 and CCL2. They also indicated that TAMs support tumor progression and metastasis by promoting angiogenesis and suppressing antitumor immune responses [8].

#### 5.2.3 Lack of Costimulatory Molecular Expression

**FIGURE 3:**
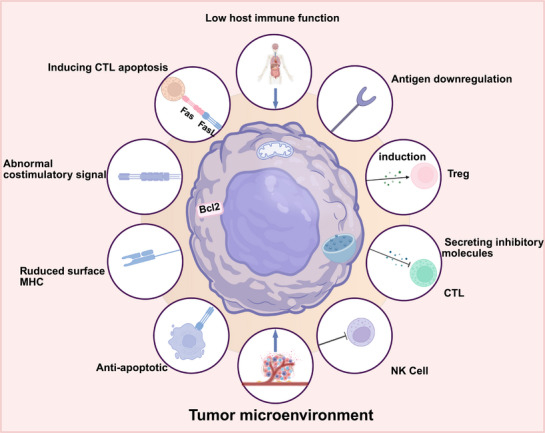
The main mechanisms associated with immune escape in tumor immunotherapy include downregulation of tumor antigen expression, alteration of MHC molecule expression, reduced immunogenicity, secretion of immunosuppressive factors, recruitment of immunosuppressive cells, and lack of costimulatory molecule expression. Created in BioRender.com.

Although tumor cells can directly present tumor antigens to T cells via MHC molecules on their surface, the absence of costimulatory signals prevents T cell activation, thereby inhibiting the effective recognition and elimination of tumor cells. This ultimately enables tumor cells to evade the immune system. For example, tumor cells may impede T cell activation and proliferation by downregulating the expression of B7 family costimulatory molecules (such as B7‐1 and B7‐2), allowing them to escape immune detection [7]. Clinically, tumors lacking costimulatory molecule expression often exhibit resistance to ICI therapy. Furthermore, some tumors evade immune responses by decreasing the expression of immune checkpoint molecules such as PD‐L1 and CTLA‐4, which can lead to immunotherapy failure [3]. This evasion mechanism enables tumor cells to continue growing and spreading even in the presence of ICIs. Additionally, a study by George et al. [132] suggests that PTEN deficiency is associated with resistance to PD‐1 checkpoint blockade therapy, likely due to its effect on costimulatory molecule expression. In the context of immune checkpoint blockade therapy, tumor cells can evade immune surveillance by reducing the expression of costimulatory molecules. Peng et al. [133] have also demonstrated that PTEN deletion enhances tumor cell resistance to T cell‐mediated immunotherapy, possibly by regulating the expression of costimulatory molecules. Figure 3 illustrates the mechanisms underlying immune evasion in tumor immunotherapy.

### 5.3 Potential Mechanisms for mRNA Vaccines to Overcome Drug Resistance

**FIGURE 4:**
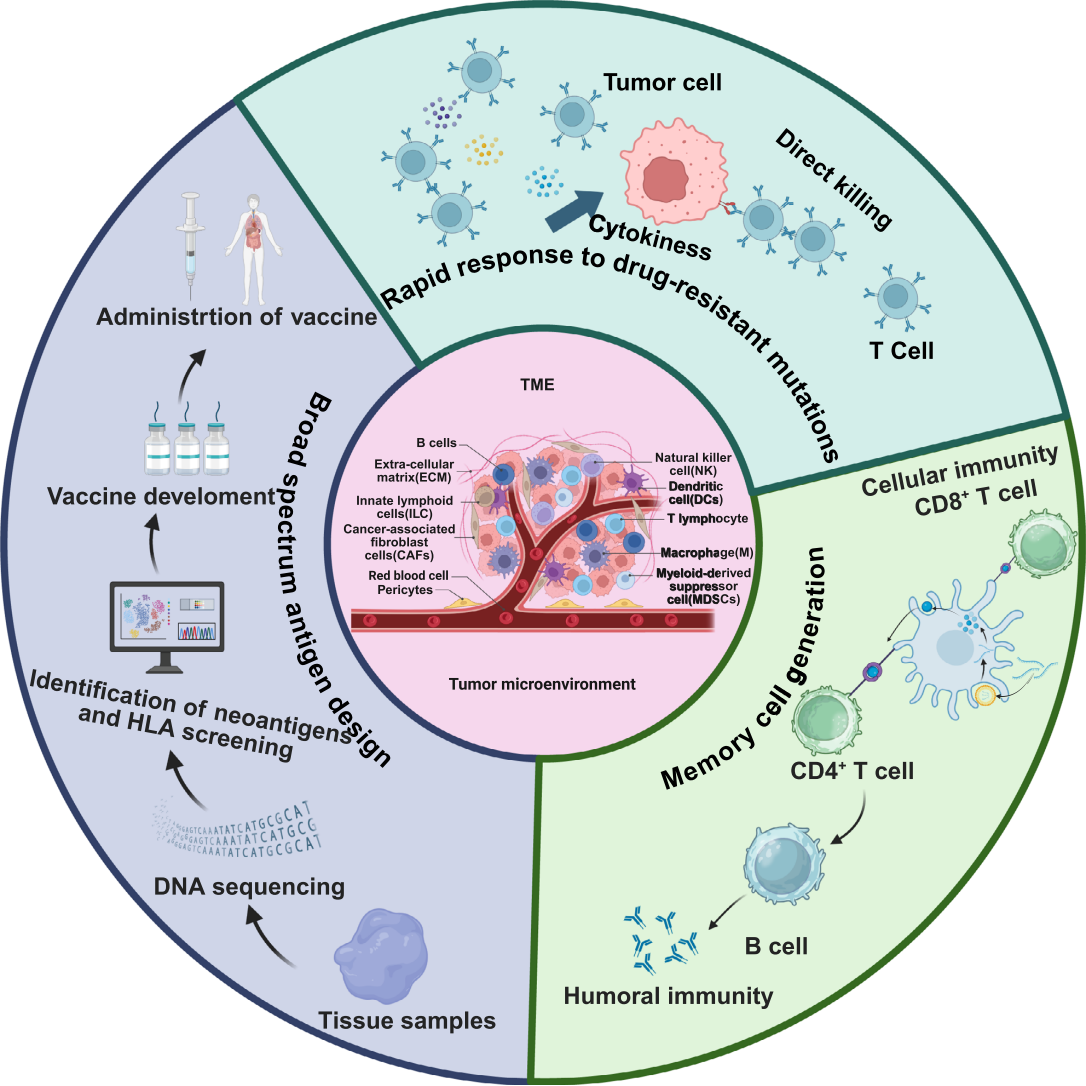
Potential mechanisms by which mRNA vaccines can overcome drug resistance. Rapid response to drug‐resistant mutations, new mRNA sequences can be rapidly designed to target drug‐resistant mutations as they arise in pathogens or tumor cells. These mRNA vaccines can induce apoptosis of tumor cells through an enhanced immune response, effectively counteracting resistance. Broad‐spectrum antigen design. By designing mRNA sequences that encode broad‐spectrum antigens, it is possible to stimulate the body's immune system to recognize and respond to multiple pathogens or tumor cell variants, thus offering more comprehensive protection. Generation of memory cells. By encoding specific antigens, mRNA vaccines effectively activate the immune system to produce memory B and T cells, resulting in long‐term immune protection against future encounters with resistant pathogens or tumor cells. Created in BioRender.com.

mRNA vaccines activate the immune system by encoding TAAs and expressing them directly within the body. Unlike conventional targeted drugs and antibody therapies, mRNA vaccines are capable of inducing a broader immune response. Targeted drugs are typically directed against specific molecular targets, while antibody therapies rely on antigen‐antibody specific binding. In contrast, mRNA vaccines can simultaneously activate multiple immune cells by encoding multiple tumor antigens, resulting in a stronger and more comprehensive immune response [134]. mRNA vaccines can also address drug resistance through several mechanisms. First, they offer rapid adaptability to drug‐resistant mutations. When drug‐resistant mutations arise in pathogens or tumor cells, researchers can quickly analyze the mutation sites and design new mRNA sequences accordingly. This flexibility enables mRNA vaccines to adapt rapidly to new mutations, thereby overcoming resistance and immune evasion issues in immunotherapy [135]. Xu et al. [136] demonstrated that mRNA vaccines could enhance T cell activity by encoding specific tumor antigens, thereby improving the immune system's ability to combat tumors. As new drug‐resistant mutations develop in tumor cells, mRNA sequences can be rapidly adjusted to maintain vaccine efficacy. Another study showed that by analyzing tumor cell mutations using whole genome sequencing and adapting mRNA sequences accordingly, therapeutic efficacy could be improved [137]. This rapid response mechanism, guided by genomic data, allows mRNA vaccines to retain high therapeutic efficiency in the face of complex tumor mutations. Second, broad‐spectrum antigen design involves engineering mRNA sequences to encode antigens that stimulate immune responses against multiple pathogens or tumor cell mutations. The key to this approach lies in identifying and selecting antigens that cover a wide range of tumor variants, thereby enhancing the effectiveness and adaptability of the vaccine. Broad‐spectrum antigen design can leverage high‐throughput sequencing to identify unique mutations within individual tumor samples, known as mutant cohorts, thus enabling personalized and comprehensive immune responses. This approach enables the rational design of tumor neoepitope vaccines tailored to individual patients, offering the advantage of specificity for nonself‐antigens that are not eliminated by central tolerance mechanisms. Nonsynonymous cancer mutations delivered via mRNA are largely immunogenic and are primarily recognized by CD4+ T cells. This personalized vaccine strategy has shown potential in controlling tumor growth in mouse models of melanoma and colon cancer. Second, mRNA vaccines can encode multiple TAAs, thereby eliciting a broader immune response. In a study by Xu et al. [136], an mRNA vaccine was synthesized containing a mixture of multiple TAAs combined with the immune‐enhancing adjuvant ImmunER, with the aim of inducing a potent antitumor immune response. This combination not only promotes DC maturation and migration but also enhances antigen presentation, thereby improving T cell proliferation and activation and ultimately leading to efficient tumor cell killing. Additionally, mRNA vaccines can overcome tumor immune escape mechanisms by encoding specific neoepitopes. Sahin et al. [96] developed personalized neoepitope mRNA vaccines and conducted clinical trials in melanoma patients. In these trials, CD4^+^ T‐cell responses to most of the neoepitopes were detected, and there was a low incidence of metastatic disease after several months of follow‐up [96]. This approach increases vaccine specificity and reduces the likelihood of tumor escape. Third, mRNA vaccines demonstrate remarkable potential for inducing robust and long‐lasting immune memory. By encoding specific antigens, mRNA vaccines effectively activate the immune system, generating memory B and T cells that provide sustained protection. For example, Zhuang et al. [138] showed that mRNA vaccines delivering H1N1 influenza virus HA proteins via cationic LNPs could induce a protective immune response in mice. This mechanism is also applicable to tumor vaccines, which deliver tumor antigens to activate DCs and elicit specific T‐cell responses. Furthermore, mRNA vaccines are capable of inducing diverse CD8^+^ T‐cell responses. Zhang et al. [134]. reported that approximately 30% of peripheral CD8^+^ T cells exhibited diverse TCR clonotypes, a diversity that persisted for at least 6 months after concluding vaccination. This heterogeneity is crucial for overcoming tumor immune evasion, as it enables recognition and targeting of various tumor antigenic epitopes. Moreover, mRNA vaccines can enhance antitumor immunity by improving the function of memory T cells, which play a significant role in optimizing tumor immunotherapy strategies, as demonstrated by Liu et al. [139]. Through mRNA vaccination, memory T cell function can be strengthened, thereby increasing the capability to eliminate tumor cells. In summary, mRNA vaccines are effective at inducing memory B and T cells by activating DCs, eliciting diverse CD8^+^ T cell responses, and enhancing memory T cell function. These memory cells are essential for long‐term protection and for overcoming tumor immune evasion. The potential mechanisms by which mRNA vaccines combat drug resistance are outlined in Figure 4.

## 6 Optimizing the Delivery and Structure of mRNA Vaccines

As an emerging nucleic acid vaccine technology, mRNA vaccines have demonstrated great potential during the COVID‐19 pandemic, owing to their advantages of rapid development, programmability, and safety. However, mRNA molecules are inherently susceptible to degradation by nucleases in vivo and exhibit poor stability during delivery and expression, as well as low translational efficiency and immunogenicity. These limitations hinder their broader application across a wider range of diseases. Therefore, optimizing both mRNA structural design and delivery systems is crucial for enhancing their efficacy and safety. In this article, we discuss recent advances in the optimization of mRNA structures and delivery systems.

### 6.1 Strategies for Optimizing mRNA Structure

#### 6.1.1 5′Cap Optimization

The cap structure is located at the 5′ end of mRNA and consists of methylated guanosine (m7G) pyro phosphorylated to the nucleotide at the 5′ terminus. During in vivo transcription, three common cap structures are observed: Cap0 (m7GpppXpYp), Cap1 (m7GpppXmpYp), and Cap2 (m7GpppXmpYmp). These cap structures significantly increase translation efficiency and enhance intracellular mRNA stability, primarily through their interaction with the eukaryotic translation initiation factor 4E [140, 141, 142]. Additionally, the absence of a terminal free phosphate group at the 5′ end after capping makes the mRNA less susceptible to degradation by alkaline phosphatase, thereby providing further protection [143, 144, 145]. Furthermore, methylated nucleotides found in Cap1 and Cap2 structures can block the free 2′‐OH group on the phosphate bond, conferring resistance to degradation by RNA enzymes. To prevent the loss of translation efficiency caused by reverse incorporation of the cap structure during IVT, researchers have developed an “antireverse” cap analogue (ARCA). By introducing 3′‐O‐methylation to the guanosine of the cap structure, ARCA ensures that the cap is incorporated in the correct orientation, thereby increasing mRNA stability and improving translation efficiency [25]. Over the years, scientists have continued to optimize ARCA, with the “CleanCap” analogue—developed by TriLink Biotechnologies in San Diego, CA, USA—now widely regarded as the most popular choice in the mRNA field [146]. Therefore, optimization of the 5' cap structure is crucial for enhancing the efficiency of mRNA vaccines and gene therapies.

#### 6.1.2 Optimization of UTRs

Optimization of UTRs is essential for effective mRNA vaccine design, as the 5′ and 3′ UTRs can significantly influence both transcription rate and mRNA half‐life [147, 148, 149]. Currently, 5' UTR sequences are often derived from genes such as β‐globin and Hsp70, or are designed de novo using big data and machine learning approaches [150, 151, 152]. To minimize ribosomal scanning and enhance translation, it is advisable to use a short 5′ UTR of at least 20 nucleotides. Potential upstream start codons, especially AUG, should be avoided to prevent the formation of upstream ORFs, which can inhibit translation. In addition, avoiding both canonical (AUG) and noncanonical (e.g., CUG) initiation codons can further optimize the 5′ UTR and prevent interference with translation initiation [153]. It is also critical to prevent the formation of stable secondary structures within the UTRs, as these can impede ribosome recruitment and codon recognition. Notably, short 5′ UTRs have been shown to enhance mRNA translation efficiency [154, 155, 156]. Bioinformatics tools can predict the translational efficiency of 5' UTR candidates, providing valuable guidance for mRNA design. Furthermore, research indicates that specific stem‐loop structures within 5′ UTRs can facilitate ribosome loading and improve translational efficiency [157]. For the 3′ UTR, novel sequence motifs have been identified that confer stronger therapeutic effects than the traditional β‐globin 3′ UTR [158, 159, 160]. In addition, incorporating AU‐ and GU‐rich elements can improve RNA stability [161, 162], while tandem repeats of the 3′ UTR can increase transcription efficiency [160]. In summary, careful optimization of UTRs is vital for maximizing translational efficiency and mRNA stability, particularly in the context of mRNA vaccines for tumor immunotherapy.

#### 6.1.3 Codon Optimization

Codons within the ORF significantly influence translation efficiency and mRNA stability. The concept of codon optimality has been introduced to address this, as the ribosome decodes different codons at varying rates, which plays a crucial role in both translation efficiency and mRNA stability [75]. Substituting nonoptimal codons with optimal ones can greatly enhance mRNA stability, accelerate the translation process, and boost protein production [163]. Additionally, codon usage frequency is closely linked to the abundance of corresponding tRNAs in host cells; optimizing codon usage can therefore improve translation efficiency and increase protein expression. Miao et al. [35] demonstrated that adjusting the guanine (G) to cytosine (C) ratio within ORFs can regulate the rate of translation elongation and reduce uracil‐rich regions, which might otherwise be recognized by RIG‐I and subsequently hinder protein expression. Codon optimization can also expedite translation by replacing rare codons and preventing the formation of stable secondary structures and hairpin loops in the ORF, which could otherwise impede proper protein folding [164, 165, 166, 167]. Moreover, Thess et al. [38, 168] found that sequence‐engineered, chemically unmodified mRNAs exhibit higher protein expression levels compared with chemically modified but noncodon‐optimized mRNAs, highlighting the importance of codon optimization in enhancing mRNA expression efficiency. In conclusion, codon optimization not only enhances the efficiency and stability of vaccine expression but also offers a promising strategy in the field of tumor immunotherapy.

#### 6.1.4 Poly(A) Tail Optimization

The poly(A) tail is a critical component of the mRNA molecule, playing a protective role in the cytoplasm by preventing degradation by exonucleases. It facilitates the assembly of the translation initiation complex through interactions with poly(A)‐binding proteins (PABPs), thereby enhancing translation efficiency [169]. Additionally, the binding of the poly(A) tail to PABPs enables efficient transport of mRNA from the nucleus to the cytoplasm. The length of the poly(A) tail significantly influences mRNA stability and translation efficiency; generally, longer poly(A) tails increase mRNA stability, although they may also affect delivery and intracellular processing in vivo. Different poly(A) tail lengths exert distinct regulatory effects on mRNA function, underscoring the importance of optimizing poly(A) tail length in mRNA design [169]. Furthermore, 2′‐O‐methylation of adenosine residues within the poly(A) tail can enhance mRNA stability and reduce immunogenicity. The function of the poly(A) tail can also be modulated by engineering specific secondary structures, such as hairpins, to further optimize mRNA performance. These strategies are essential for improving mRNA stability, translational efficiency, and therapeutic efficacy in applications such as tumor immunotherapy.

### 6.2 Development and Optimization of Delivery Systems

#### 6.2.1 Lipid Nanoparticles

Originally designed for siRNA delivery, ionizable LNPs have become the most widely used mRNA delivery system today [170, 171, 172]. Since the feasibility of in vivo translation of mRNA‐LNPs was demonstrated in 2015 [173], numerous vaccine studies have utilized both unmodified and nucleoside‐modified mRNAs encapsulated in LNPs, resulting in the successful induction of long‐term protective immune responses against a wide range of infectious pathogens [174, 175, 176, 177, 178, 179, 180, 181]. Additionally, mRNA‐LNPs have shown significant efficacy in the field of anticancer therapy [182]. Several clinical trials using mRNA‐LNPs are ongoing, and published data from two phase I influenza virus vaccine trials (NCT03076385 and NCT03345043) are already available [54, 174]. LNPs are primarily composed of three main components: ionizable lipids (40–50%), cholesterol (38–45%), and auxiliary phospholipids (10–12%). In some cases, a fourth component, such as polyethylene glycol (PEG)‐conjugated lipids (1–2%), is included [183, 184]. These components together encapsulate and protect the naked mRNAs [185]. LNPs not only shield mRNAs from degradation in vivo but also promote their efficient intracellular delivery and expression. The choice of lipid type is a key strategy for optimizing LNP performance, as different lipid materials can greatly influence particle stability and delivery efficiency. For instance, several studies have shown that the use of unsaturated lipids improves mRNA transfection efficiency in vivo [186]. Additionally, certain lipid combinations can activate STING‐mediated immune responses, thereby enhancing antitumor effects [187]. The control of particle size is equally important for optimizing LNP performance. Studies have demonstrated that LNP particle size directly affects their in vivo distribution and cellular uptake efficiency. Smaller nanoparticles are more readily endocytosed by cells, which increases mRNA delivery efficiency [188]. Furthermore, the particle size of LNPs influences their biodistribution across different tissues and thus their therapeutic outcomes. For example, adjusting LNP particle size can facilitate targeted delivery to specific organs [189]. To further enhance LNP performance, researchers have explored various chemical modification strategies. For example, surface modification with PEG can significantly prolong LNP circulation time in vivo and improve stability [35]. In addition, novel amino lipid materials have been developed to promote endosomal escape, thereby improving both the efficiency and safety of mRNA delivery [154]. The use of these strategies offers great promise for advancing LNPs in mRNA vaccine delivery.

#### 6.2.2 Polymer Carriers

**FIGURE 5:**
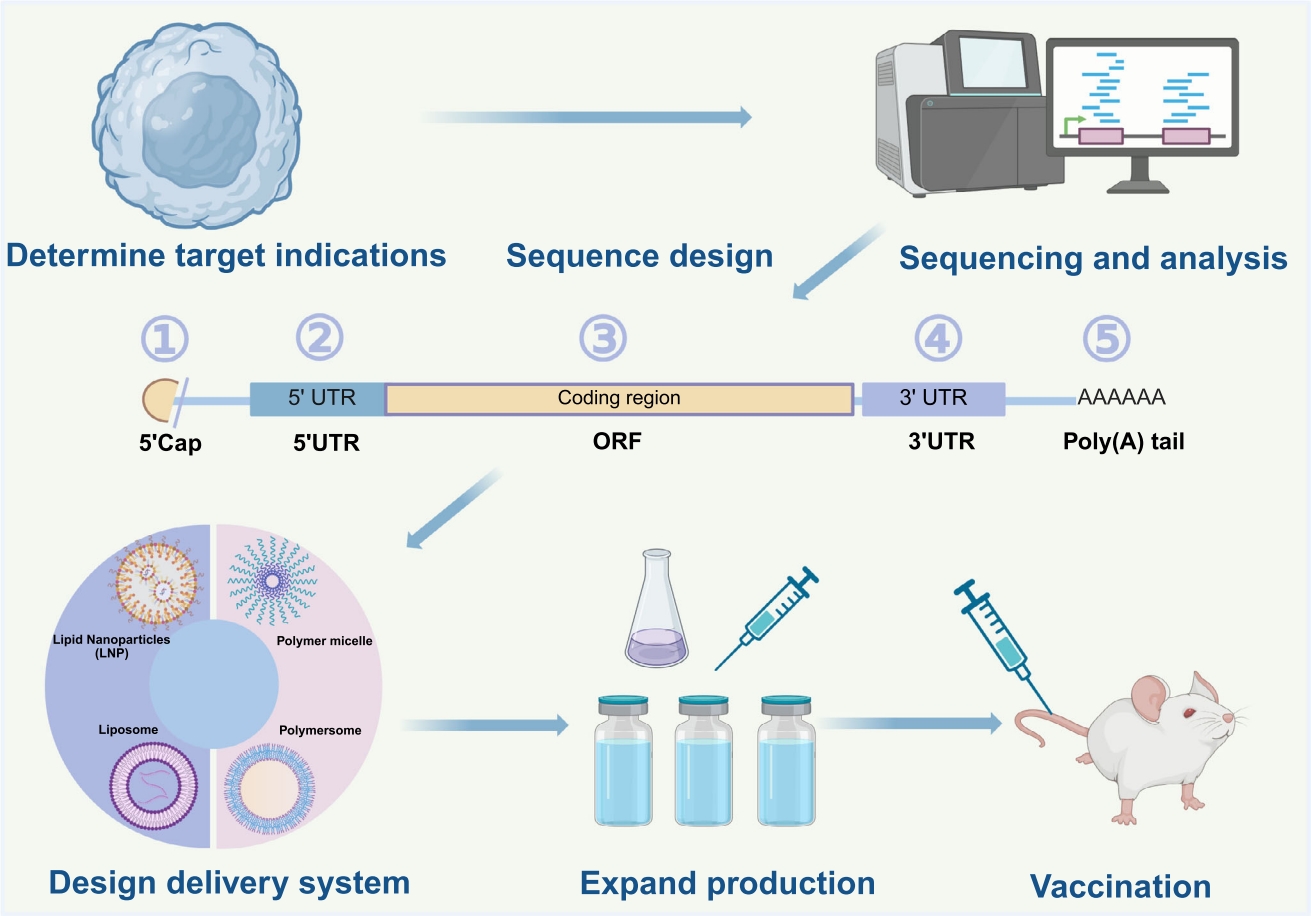
Patient tumor tissue specimens were first collected for whole exome sequencing. Next, machine learning‐based MHC affinity ranking AI was used to predict and screen for potential tumor neoantigens or tumor‐associated antigens. These findings guided the sequence design of the mRNA vaccine. The development process included the following key steps: ① (1) Developing ARCA capping. (2) Reducing sensitivity to decapping enzymes. ② Inserting specific sequences. ③ Replacing rare codons with more frequently used codons. ④ Incorporating random sequence stabilization software. ⑤ Slowing down the degradation process by RNA exonucleases. Subsequently, the mRNA vaccine was delivered using lipid nanoparticles (LNPs) and polymers. Finally, the mRNA vaccine was formulated and administered by injection. Created in BioRender.com.

Cationic polymers offer significant flexibility for structural modification and development, enabling the condensation of negatively charged mRNAs through electrostatic interactions. This protects mRNA from nuclease degradation and ultimately enhances delivery efficiency. Currently, cationic polymers such as polyethyleneimine (PEI), poly(β‐amino esters) (PBAE), chitosan, and others are commonly employed for mRNA delivery; these often require further modification to boost transfection efficiency and stability [190, 191, 192]. Recent research has focused on optimizing polymeric carriers to improve the efficacy of mRNA vaccines through various strategies [193]. First, the choice of polymer type is crucial for effective mRNA delivery. For example, combining low molecular weight PEI with cyclodextrins has been shown to be a safe and efficient method for mRNA delivery, yielding differing antibody responses depending on the route of administration [194, 195]. Additionally, researchers have developed libraries of poly(amino esters) for structure–function studies, highlighting their potential for pulmonary mRNA delivery [196, 197]. Surface modification is another key factor in optimizing polymeric carriers. For instance, the hydrogen bonding between PEGylated oligonucleotides and mRNA can significantly improve both stability and delivery efficiency [186]. Furthermore, the charge density and molecular weight of polymer carriers impact their delivery effectiveness, often necessitating modifications to further enhance transfection efficiency [198, 199, 200, 201, 202, 203, 204]. Moreover, pH‐responsive polymers—which degrade in response to cytoplasmic pH changes—have been explored to facilitate intracellular mRNA release [205]. Amphiphilic polymers containing cationic or amphiphilic amine groups have also demonstrated promise for forming nanocomplexes with mRNA, thereby improving delivery efficiency [187]. A summary of sequence and vector optimization strategies for tumor mRNA vaccines is provided in Figure 5.

## 7 Conclusions and Future Prospects

As an emerging immunotherapeutic tool, mRNA vaccines have demonstrated great potential in controlling the COVID‐19 outbreak. Compared with conventional vaccines, mRNA vaccines offer several advantages, including rapid development, high production flexibility, and the ability to target multiple antigens. They also exhibit a favorable safety profile, with side effects generally limited to transient local reactions or mild systemic symptoms. Furthermore, mRNA vaccines do not require the use of viral vectors or intact viruses, thereby reducing the risks associated with viral components. However, despite the remarkable success of mRNA vaccines in the field of antiviral therapy, several challenges remain before their widespread adoption can be fully realized. Issues such as vaccine stability, delivery efficiency, durability of the immune response, and cost still require further optimization, particularly regarding the molecular structure of mRNA and the development of effective delivery vectors. Recent studies have shown that the efficacy of mRNA vaccines depends not only on the encoded antigen but also on the structure of the mRNA and the delivery system employed. Structural optimization of mRNA molecules can enhance their stability and immunogenicity in vivo. Additionally, the choice and refinement of delivery vehicles are critical to the success of mRNA vaccines, as appropriate delivery systems can ensure efficient mRNA transport to target cells and boost the overall effectiveness of the immune response

Currently, research on the molecular pharmacological mechanisms of mRNA vaccines remains limited. It is hoped that future studies will focus on how mRNA vaccines affect various immune cell populations including DCs, B cells, and T cells particularly regarding their roles in regulating innate and adaptive immune responses. Furthermore, investigations into the immune tolerance and immune escape mechanisms associated with mRNA vaccines should also be prioritized, especially with the aim of enhancing the broad‐spectrum protective effects of these vaccines against mutated viruses.

Looking ahead, mRNA tumor vaccines are expected to become an integral component of comprehensive cancer therapy, complementing traditional approaches such as surgery, chemotherapy, radiotherapy, and ICIs. These vaccines promise to offer patients more diverse and effective treatment options. With the ongoing advancement of personalized medicine, mRNA tumor vaccines have the potential to revolutionize cancer care, significantly improving patient survival rates and quality of life. In the future, research should focus on the integrated use of advanced technologies such as machine learning, AI, and deep learning algorithms to analyze and predict the performance of various delivery vehicles including lipids, polymers, and inorganic nanoparticles. This approach will help identify their immunological effects and optimize their stability and targeting within the body. Moreover, these technologies will enable researchers to rapidly design mRNA molecules with enhanced stability, higher translational efficiency, and reduced degradation in vivo. It is anticipated that, through the persistent efforts of researchers, mRNA cancer vaccines will continue to be optimized, bringing greater hope to cancer patients and becoming a powerful tool in the fight against cancer.

## Author Contributions

Jianmei Li, Yixin Liu, and Jie Dai prepared the figures and the manuscript, including searching the literature and writing the original draft and editing. Li Yang, Feng Xiong, Jing Xia, and Jing Jin revised the details of this review. Yangping Wu and Xingchen Peng edited the manuscript. All authors have read and approved the final manuscript.

## Conflicts of Interest

The authors declare no conflicts of interest.

## Ethics Statement

The authors have nothing to report.

## Data Availability

The authors have nothing to report.
